# The Role of Intra-aortic Balloon Pump Therapy at Resource-Limited Institutions: A Bridge to Care Escalation

**DOI:** 10.7759/cureus.100844

**Published:** 2026-01-05

**Authors:** Ricky Patil, Eric Chuang, Fareed Cheema, Sunil V Rao, Mikhail Vaynblat

**Affiliations:** 1 Department of Surgery, NYU Langone Health, Brooklyn, USA; 2 Department of Cardiology, NYU Langone Health, Brooklyn, USA; 3 Department of Cardiothoracic Surgery, NYU Langone Health, Brooklyn, USA

**Keywords:** hub-and-spoke hospital system, intraaortic balloon pump, patient transfer, resource limitations, stemi

## Abstract

Despite providing relatively modest circulatory support, the intra-aortic balloon pump (IABP) remains the most utilized mechanical support device. IABP therapy specifically provides utility in transferring critically ill patients from resource-limited hospital settings. In a single-center series, 71 patients who received IABP were identified from 2018 to 2023. In this group, 66 (93%) patients presented with acute myocardial infarction (AMI), of which 40 (61%) patients presented with STEMI. Sixty-three (89%) patients presented in cardiogenic shock. In total, 15 (21%) patients died during their hospital stay. In-hospital death was found to be associated with higher age (p < 0.001), female sex (p = 0.004), and chronic heart failure (p = 0.009). Serologic markers of end-organ perfusion, such as lactate, creatinine, and hepatic enzymes, were associated with increased mortality risk. Thirty (41%) patients were successfully transferred to a hub institution for care escalation, including 17 (57%) patients receiving cardiac surgery, five (17%) receiving advanced PCI, and 12 (40%) receiving more robust mechanical support. Therefore, in the real-world setting, IABP therapy provides an accessible form of circulatory support at resource-limited institutions, especially when patient transfer is required within a larger hospital system.

## Introduction

The intra-aortic balloon pump (IABP) is the most utilized mechanical circulatory support (MCS) device. However, the advent of more robust MCS technology and the lack of demonstrated survival benefit in prospective literature have called into question its role in contemporary practice [1-3]. Several observational studies have shown decreases in the use of IABP since the publication of the IABP-SHOCK II trial, which showed no survival benefit with routine IABP use in patients with acute myocardial infarction (MI)-related cardiogenic shock receiving early revascularization therapy [4,5]. In addition, clinical guidelines have incorporated these data and have downgraded the routine use of IABP for shock, but maintain a class 2A recommendation for acute coronary syndrome (ACS)-related mechanical complications as a bridge to surgery [6]. Similarly, the 2023 European Society of Cardiology (ESC) guidelines give a class 3 recommendation against the routine use of IABP in acute myocardial infarction (AMI) with cardiogenic shock, but maintain a class 2A recommendation for IABP use in heart failure secondary to mechanical complications of ACS, albeit based on Level C evidence [7].

However, hospitals lacking infrastructure or expertise in more advanced mechanical support may rely uniquely on IABP therapy, a niche not well-captured in the existing literature. For such hospitals that serve as “spoke” facilities in a “hub-and-spoke” system, the IABP may provide an easily placed temporary support device that facilitates safe transfer for a higher level of care. Additionally, while IABP provides limited mechanical support, its deployment and management are associated with a lower complication rate compared to larger bore devices such as the micro axial flow pump [8]. The current study, conducted at a hospital without on-site cardiopulmonary bypass (CPB) capabilities or a more advanced circulatory support device, is a descriptive series that aims to characterize the indications and outcomes of IABP use in a resource-limited setting, including its role as bridge therapy during transfer to tertiary-care facilities. We hypothesize that in the community setting, IABP therapy provides an accessible form of mechanical support when escalation of care is required.

This article was previously presented as a meeting abstract at the 2025 International Society of Minimally Invasive Cardiac Surgery (ISMICS) Annual Meeting on May 30th, 2025.

## Materials and methods

Sample

All patients who underwent IABP therapy at a single community hospital between February 2018 and July 2023 were identified through chart review. All adult patients who had an IABP placed in the hospital’s cardiac catheterization laboratory were included in the analysis. Patients who received an IABP after transfer to another facility were excluded from further evaluation. All patients who received percutaneous coronary intervention (PCI) for ACS were treated with dual antiplatelet therapy. In patients requiring coronary artery bypass graft (CABG), only aspirin mono-therapy was continued. In preparation for PCI, patients were anti-coagulated to an ACT goal of 250 with either intravenous heparin or bivalirudin. Anti-coagulation was continued in patients with prior indication (i.e., atrial fibrillation) or in select cases where prolonged IABP therapy was anticipated. Selection of anti-coagulation and anti-platelet agents and dosage was left to the discretion of the practitioner based on evaluation of bleeding and thrombosis risk. 

This study was done at a satellite institution with resource limitations, which was defined by a lack of access to CPB, on-site cardiac surgery expertise, and/or cardiothoracic intensive care unit (ICU) amenities. Additionally, more advanced mechanical support devices, such as the micro-axial flow pump, temporary ventricular assist devices (VAD), and/or extracorporeal membrane oxygenation (ECMO), were not available on-site at this institution. While on-site PCI with door-to-balloon time < 90 minutes was available, more complex percutaneous procedures needed referral to a larger, flagship institution. Transferred patients were relocated approximately 12 miles in an ambulance through a large metropolitan area from one institution to another. Time of transfer ranged from 30 minutes to one hour but varied greatly based on time of day, bed availability, and case urgency. 

Outcomes

Comorbidity burden was documented based on admission history and physical documentation. IABP placement indications, hemodynamics, and extent of coronary artery disease were obtained from index cardiac catheterization reports. The primary outcomes studied are (1) incidence of successful transfer to a hub institution and (2) overall in-hospital mortality. Secondary outcomes include pre-IABP metrics such as cardiac disease etiology, hemodynamics, and serology, as well as post-procedural outcome measures such as treatment destination, time on IABP therapy, and hospital length of stay. For hemodynamic evaluation, the lowest mean arterial pressure (MAP) and systolic blood pressure (SBP) in the first 24 hours of presentation were included. On evaluation of lab markers, peak lactate, creatinine, hepatic enzymes, as well as the lowest hemoglobin level and platelet count in the first 24 hours of presentation, were included in the analysis. Lastly, the number of vasopressors and inotropes required at the time of IABP deployment was also included. Cardiogenic shock was defined as the need for vasopressor support, a sustained SBP < 90 mmHg, or a pre-procedural lactate > 2 mmol/L. 

Analysis

Survivors and in-hospital mortalities were compared using Fisher’s exact test and the Wilcoxon rank-sum test for categorical and continuous variables, respectively. Categorical data were presented as count (n%), and continuous data were expressed as a median (Q1, Q3). Kaplan-Meier analysis was conducted to evaluate in-hospital survival, with censoring at patient discharge. Statistical analysis was done with the Stata/SE 18 software package (StataCorp LLC, College Station, TX).

## Results

**Figure 1:**
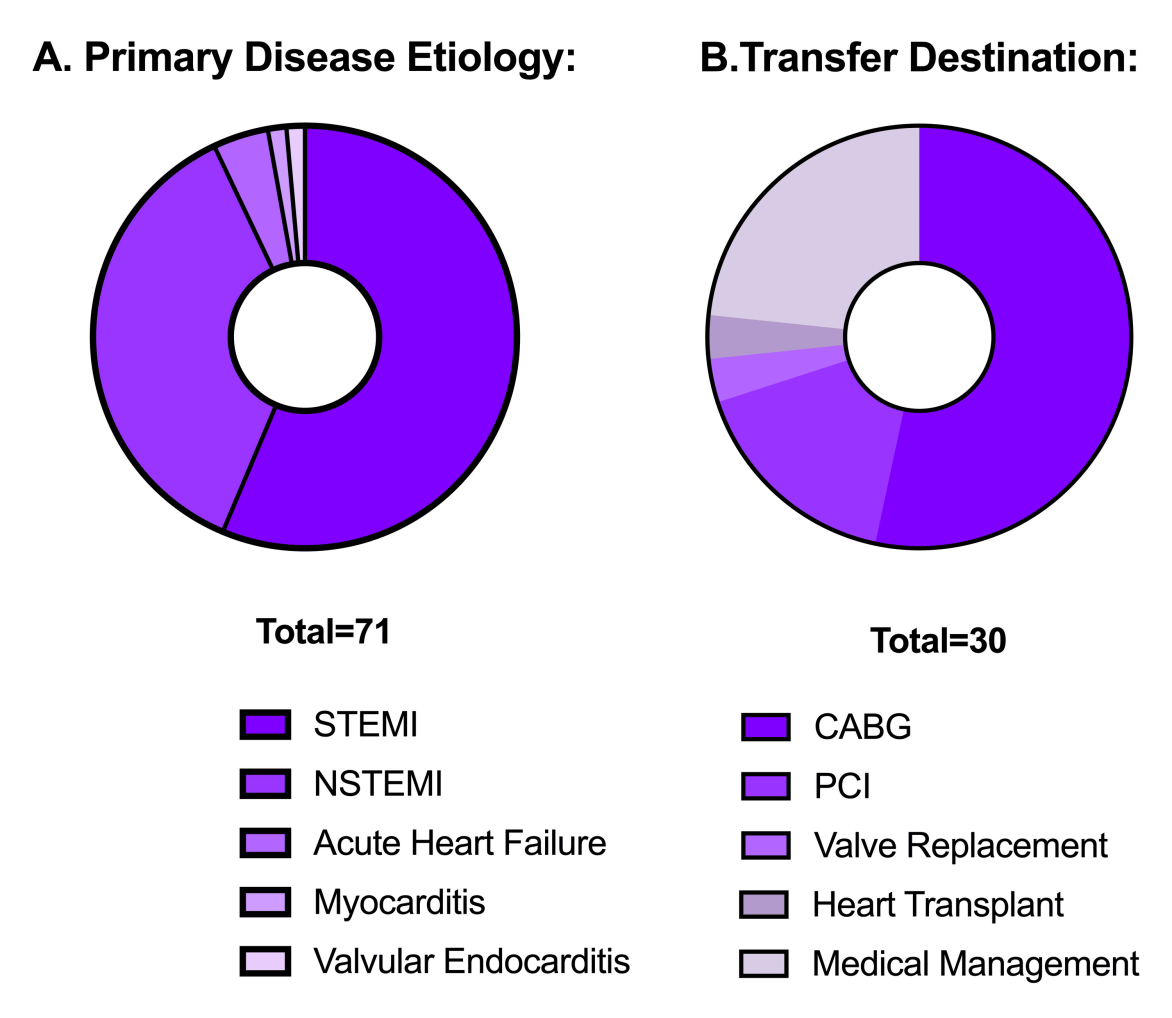
Overall disease etiology and treatment destination for transferred patients (A) Breakdown of patient disease etiology: STEMI: 40 (56%), NSTEMI: 26 (37%), acute heart failure: 3 (5%), myocarditis: 1 (1%), valvular endocarditis: 1 (1%). (B) Treatment destination for transferred patients: CABG: 16 (53%), advanced PCI: 5 (17%), valve replacement: 1 (3%), heart transplant: 1 (3%), medical management: 7 (24%). All 41 non-transferred patients were bridged to myocardial recovery without secondary intervention (not shown). CABG, coronary artery bypass grafting; NSTEMI, non-ST-elevation myocardial infarction; PCI, percutaneous coronary intervention; STEMI, ST-elevation myocardial infarction

We identified 71 patients who received an IABP that met selection criteria (Table 1). Sixty-six (93%) patients presented with AMI, including 40 (56%) patients with ST-segment elevated MI (STEMI) and 26 (37%) with non-ST-segment elevated MI (N-STEMI). Among patients with AMI, four (6%) patients required IABP placement pre-PCI for impending hemodynamic collapse, while the remainder received IABP after initial angiography. The remaining five (7%) patients presented with non-AMI conditions, including three (4%) cases of non-ischemic heart failure exacerbation, one (1%) case of sterile myocarditis, and one (1%) case of bacterial mitral valve endocarditis. Sixty-three (89%) of patients presented in shock (Figure 1A).

**Table 1:** Patient characteristics Demographics, relevant medical history, select measurements from index catheterization, and short-term outcomes after IABP implantation, for categorical analysis via. Fisher’s exact test, the Pearson chi-squared test statistic was provided. ^#^For analysis of continuous variables via. Wilcoxon rank-sum test, the z-score was provided. ^^^Only 48/71 patients received admission arterial lactate values, and only 63/71 patients received admission AST and ALT values. IABP, intra-aortic balloon pump; MAP, mean arterial pressure; MI, myocardial infarction; NSTEMI, non-ST-elevation myocardial infarction; SBP, systolic blood pressure; STEMI, ST-elevation myocardial infarction

Demographic Information	All Patients (71)	Survivors (56)	In-Hospital Mortality (15)	Test Statistic	Significance
Female Sex (%)	20 (28%)	11 (20%)	9 (60%)	9.5	0.004
Age (Years)	65 (57, 75)	61 (53, 70)	76 (69, 85)	-3.8^#^	<0.001
Comorbidity Burden					
Chronic Heart Failure	33 (49%)	23 (41%)	12 (80%)	7.2	0.009
Chronic Kidney Disease	16 (23%)	12 (21%)	4 (27%)	0.2	0.73
Diabetes	30 (42%)	24 (43%)	6 (40%)	<0.1	1.0
Hypertension	43 (61%)	34 (61%)	9 (60%)	<0.1	1.0
Hyperlipidemia	30 (42%)	23 (41%)	7 (47%)	0.2	0.8
Current Smoker	26 (37%)	22 (39%)	4 (27%)	0.8	0.5
Cardiovascular Characteristics					
Disease Etiology					
STEMI	40 (56%)	31 (55%)	9 (60%)	0.1	0.8
NSTEMI	26 (37%)	21 (38%)	5 (33%)	<0.1	1.0
Non-MI Pathology	5 (7.0%)	4 (7.1%)	1 (7%)	<0.1	1.0
Triple Vessel Disease	29(41%)	25 (45%)	4 (27%)	1.6	0.2
Left Main Disease	13 (18.3%)	9 (16%)	4 (27%)	0.9	0.5
Prior Percutaneous Coronary Intervention (PCI)	18 (25%)	15 (27%)	3 (20%)	0.3	0.7
Ejection Fraction (%)	35 (20, 45)	35 (25, 47)	25 (17, 40)	1.4^#^	0.2
Hemodynamics					
Systolic Blood Pressure (SBP) (mmHg)	96 (83, 110)	97 (88, 110)	91 (68, 116)	0.57^#^	0.57
Mean Arterial Pressure (MAP) (mmHg)	70 (64, 78)	71 (65, 79)	68 (58, 72)	1.8^#^	0.07
Cardiogenic Shock	63 (89%)	49 (88%)	14 (93%)	0.5	1.0
Elevated Left Ventricular End Diastolic Pressure (>12 mm Hg)	47 (66%)	38 (68%)	9 (60%)	0.3	0.6
Number of Vasopressors/Inotropes Required at IABP Implantation	0 (0, 2)	0 (0, 1)	2 (1,3)	-4.2^#^	<0.001
Admission Laboratory Values					
Lactate (mmol/L)^	2.5 (1.7, 4.2)	2.3 (1.6, 3.3)	7 (2.1, 14.2)	-2.3^#^	0.02
Creatinine (mg/dL)	1.9 (1, 2.2)	1.4 (0.9, 1.9)	2.1 (1.3, 2.7)	-2.4^#^	0.02
Aspartate Aminotransferase (AST) (U/L)^	91 (29, 410)	67 (27, 293)	374 (74, 983)	-2.6^#^	0.01
Alanine Aminotransferase (ALT) (U/L)^	53 (28, 111)	44 (23, 91)	101 (53, 222)	-1.9^#^	0.05
Hemoglobin (g/dL)	12.9 (11.6, 14.3)	13.2 (11.9, 14.4)	11.6 (10, 12.8)	2.4^#^	0.02
Platelet Count (×10^3^/mL)	227 (164, 273)	229 (184, 275)	212 (141, 268)	1.2^#^	0.25
IABP Metrics					
Duration of IABP Therapy (Days)	3 (2 ,5)	3 (2, 5)	2 (1, 3)	1.5^#^	0.1
Outcomes					
Length of Stay (Days)	7 (4, 16)	7 (4.5, 16.5)	3 (1, 7)	2.8^#^	0.004
Transfer to Tertiary Care Center	30 (41%)	27 (48%)	3 (20%)	3.9	0.08
Bridged to CABG	16 (23%)	16 (29%)	0 (0%)	5.5	0.02
Bridged to Heart Transplant	1 (1%)	1 (1%)	0 (0%)	0.3	1.0
Bridged to Advanced PCI	5 (7%)	4 (7%)	1 (7%)	0.7	0.4
Bridged to Valve Surgery	1 (1%)	1 (1%)	0 (0%)	0.3	1.0
Bridge to Other Circulatory Support	12 (17%)	9 (16%)	3 (20%)	0.1	0.7

Thirty patients (41%) required transfer to a tertiary care center for care escalation, of which 16 (53%) underwent CABG, one (3%) received a heart transplant, one (3%) underwent mitral valve replacement, five (17%) received advanced PCI, and seven (20%) were managed medically (Figure 1B). Of all patients, 12 (17%) were escalated to other support devices; one (1%) received venoarterial (VA) extracorporeal membrane oxygenation (ECMO), and 11 (20%) received percutaneous micro-axial pump implantation. The 41 (59%) patients not transferred remained on IABP post-PCI as a bridge to myocardial recovery.

**Figure 2:**
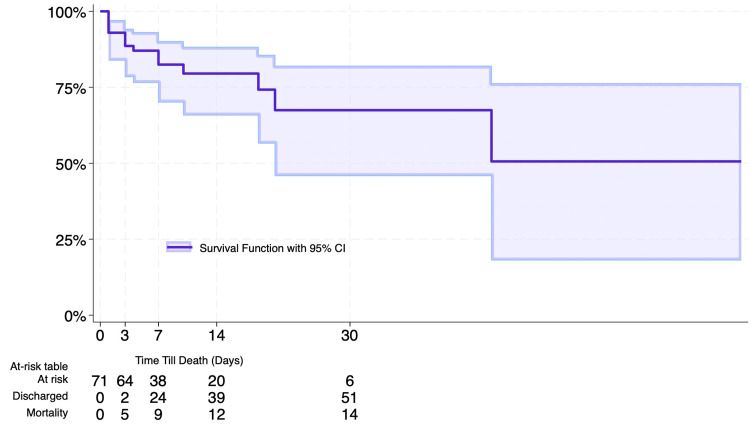
In-hospital survival analysis Kaplan-Meier curve of inpatient survival (days), censored at patient discharge.

Of the 71 patients reviewed, 56 (79%) survived their index hospitalization, while 15 (21%) patients died during their hospital stay. Patients who died were significantly older than survivors (p < 0.001), were more often female (p = 0.004), and had a higher prevalence of chronic heart failure (p = 0.009). Additionally, fatalities had higher admission peak lactate, creatinine, AST, and ALT values and lower hemoglobin count compared to survivors (p < 0.05 for all). Survival analysis demonstrated survival rates of 89% at hospital day 4 and 67% at hospital day 30. Five (33%) mortalities occurred by hospital day 3. After seven days, nine (60%) in-hospital mortalities had occurred, and 24 (42%) of survivors had been successfully discharged (Figure 2). Lastly, 11 (73%) mortalities presented in Society for Cardiovascular Angiography and Interventions (SCAI) Stage E, suggesting that a determinant of in-hospital mortality was advanced cardiogenic shock that was unable to be stabilized upon presentation. In fact, survivors had significantly higher lengths of stay compared to mortalities, driven by the inability to stabilize mortalities on initial presentation (p = 0.004). 

Lastly, patients demonstrated a high rate of in-hospital morbidity. Specifically, two (3%) patients experienced brain injury, of which both died (χ² = 7.7, p = 0.04). Thirteen (18%) patients experienced cardiac arrest, of which nine patients died (χ² = 22, p < 0.001). Lastly, nine (13%) patients experienced access site injuries, of which two patients died (χ² < 0.1, p = 1.0). Access site injuries included four cases of direct arterial injury with excessive hemorrhage, two cases of acute limb ischemia, two cases of arterial pseudo-aneurysm, and one case of iatrogenic arterial venous fistula formation.

## Discussion

This series of IABP usage at a satellite institution reflects how IABP is utilized in the community setting. IABP therapy was successfully used as temporary support toward myocardial recovery or care escalation via patient transfer. Previous studies have demonstrated poorer outcomes for patients with cardiogenic shock at satellite institutions [9]. However, the advent of protocolized approaches allows for swift triage and escalation of patient care to better-equipped institutions. This series showed a near 80% inpatient survival, thus demonstrating the improved outcomes associated with modern care. Thus, IABP therapy provides an important adjunct for patients being treated within the “hub-and-spoke” model by providing an easily managed form of temporary support. This separates IABP therapy from other MCS modalities, where cost and treatment complexity preclude usage at resource-limited institutions.

In the age of early re-vascularization therapy, the role of IABP in AMI management remains disputed. The IABP-SHOCK II trial definitively showed no survival benefit with IABP therapy in the treatment of AMI-associated cardiogenic shock treated with early re-vascularization on both short and long-term follow-up [2,3]. Thirty-day mortality was near 40% in this trial, almost double that of our present analysis, suggesting that outcomes may have improved with modern care. However, 11% of patients in our study did not meet criteria for shock, indicating that a healthier patient cohort also may have contributed to improved survival outcomes. Similarly, recent registry-level data have demonstrated a survival benefit with IABP therapy in the treatment of patients with milder shock states, but not those with more advanced stages [10]. In our series, the majority of in-hospital mortalities presented in SCAI Stage E. Thus, prudent appraisal of disease severity is key prior to IABP implantation. 

Our findings suggest that IABP therapy may fulfill a unique niche in patients requiring temporary support during hospital transfer. More than half of patients treated for cardiogenic shock at hub institutions present from another hospital, thus representing a large subset of patients in need [11]. Analysis of 1,890 patients from the national Cardiogenic Shock Working Group registry identified similar 30-day mortality between transferred and non-transferred acute myocardial infarction-cardiogenic shock (AMI-CS) patients. Almost 20% of patients transferred for treatment of AMI-CS received isolated IABP therapy, representing the most utilized MCS device in the study. Additionally, pre-transfer or early MCS implantation was identified as an independent predictor of improved survival (p = 0.046) [12]. While a more focused study is required, early mechanical support may reduce myocardial oxygen demand in the acute setting and promote better outcomes in transferred patients.

Identifying mortality predictors during IABP therapy is important to optimizing patient selection. A sub-study of the IABP-SHOCK II population identified age > 73, prior stroke, admission glucose over 191 mg/dL, creatinine > 1.5 mg/dL, thrombolysis in myocardial infarction (TIMI) score < 3, and admission lactate > 5 mmol/L as independent predictors of 30-day mortality on multi-variable Cox regression analysis [13]. Similarly, greater age, admission lactate, and creatinine were also found to be associated with mortality in our analysis. Interestingly, an association between 30-day mortality and female sex was also appreciated. Post hoc analysis of the IABP-SHOCK II trial showed that women presented with a greater comorbidity burden and had higher mortality at hospital day 1. However, when adjusted for age and other risk factors, sex was not a significant determinant of mortality [14]. Further study into the effects of sex on IABP therapy is warranted. Lastly, chronic heart failure was the only co-morbidity associated with mortality in our experience. While larger studies are required to validate these findings, this may indicate that patients with chronically diseased hearts may need more robust support than what the IABP can provide. 

This study has several limitations. First, as a retrospective study with a limited sample size, the findings discussed are limited in generalizability. Additionally, larger multivariate analyses are better equipped to identify independent predictors of mortality. This study was only capable of providing univariate associations due to the small study population. Next, the effects of selection bias should be acknowledged. The decision to place an IABP was left to the discretion of the interventional cardiologist. Hence, patients who are not IABP candidates are not represented in this study. Furthermore, transferred patients were probably more hemodynamically stable than the entire cohort, thus allowing for safe transfer. Hence, survivor outcomes and transfer status are skewed by patient selection. Overall, this study does not demonstrate the efficacy or quality of IABP's hemodynamic support. Instead, the findings documented aim only to describe the use of IABP therapy in the real-world setting as a vehicle for patient stabilization and hospital transfer. Further exploration is required to better contextualize the presented findings.

## Conclusions

In conclusion, despite providing modest MCS, the IABP still remains an important tool in the modern clinician's armamentarium. IABP therapy specifically provides care for patients needing temporary support during hospital transfer. While other therapies may offer greater hemodynamic support, the IABP remains an accessible and manageable option for therapy at resource-limited institutions that do not have access to more advanced devices. Specifically, IABP was successfully used to bridge patients to myocardial recovery, greater mechanical support, and/or definitive intervention. This series shows a near 80% survival rate among patients requiring IABP support, demonstrating an improvement from historical outcomes. Patients presenting with advanced shock stages or baseline cardiomyopathy may be at higher mortality risk. Hence, further elaboration on determinants of IABP candidacy in a modern, real-world setting is warranted. Overall, IABP remains a staple therapy in the community setting during times of treatment escalation.
